# Mixed cranial neuropathies due to occult perineural invasion of basal cell carcinoma

**DOI:** 10.1016/j.ajoc.2018.12.018

**Published:** 2018-12-19

**Authors:** Davin C. Ashraf, Evan Kalin-Hajdu, Marc H. Levin, Robert C. Kersten

**Affiliations:** a Department of Ophthalmology, University of California, San Francisco, 10 Koret Way, San Francisco, CA, 94143, USA; b Department of Ophthalmology, Palo Alto Medical Foundation, 795 El Camino Real, Palo Alto, CA, 94301, USA

**Keywords:** Basal cell carcinoma, Perineural invasion, Cranial neuropathy, BCC, Perineural spread

## Abstract

**Purpose:**

To report a diagnostically challenging case of cranial neuropathy due to perineural invasion by a basal cell carcinoma presenting 7.5 years after treatment of the primary tumor with Mohs micrographic surgery.

**Observations:**

A 62-year-old male with a history of Mohs micrographic surgery for basal cell carcinoma (BCC) of the left brow presented with insidious onset of diplopia and paresthesia localizing to the ipsilateral cranial nerves V_1_, V_2_, and VI. He had no evidence of recurrent cutaneous BCC. Magnetic resonance imaging of the orbits and skull base identified equivocal, subtle abnormalities in the ipsilateral superior orbital fissure and cavernous sinus, with normal appearance of the clinically involved nerve branches. A radiographically normal branch of cranial nerve V was biopsied and histopathology identified perineural invasion by recurrent basal cell carcinoma.

**Conclusions and importance:**

The diagnosis of perineural invasion by BCC can pose several challenges, including subtle to absent imaging findings of clinically involved nerves and a lengthy latent period following primary tumor treatment. This case represents, to our knowledge, the longest reported interval between primary treatment and biopsy-proven recurrence with perineural invasion by BCC.

## 1 Introduction

Cutaneous malignancies have a well-reported propensity to invade the perineural sheath of adjacent nerve fibers.^1^, ^2^, ^3^, ^4^ Perineural invasion (PNI) allows the contiguous spread of malignant cells along the course of the involved nerve(s). When a large branch is affected, symptomatic neuropathy may develop. The timely diagnosis of cranial neuropathy due to PNI can prove challenging due to several factors. This report describes the diagnostic challenges encountered for a patient with a remote history of Mohs-excised basal cell carcinoma (BCC) who developed progressive cranial neuropathies due to PNI.

## 2 Case report

**Fig. 1:**
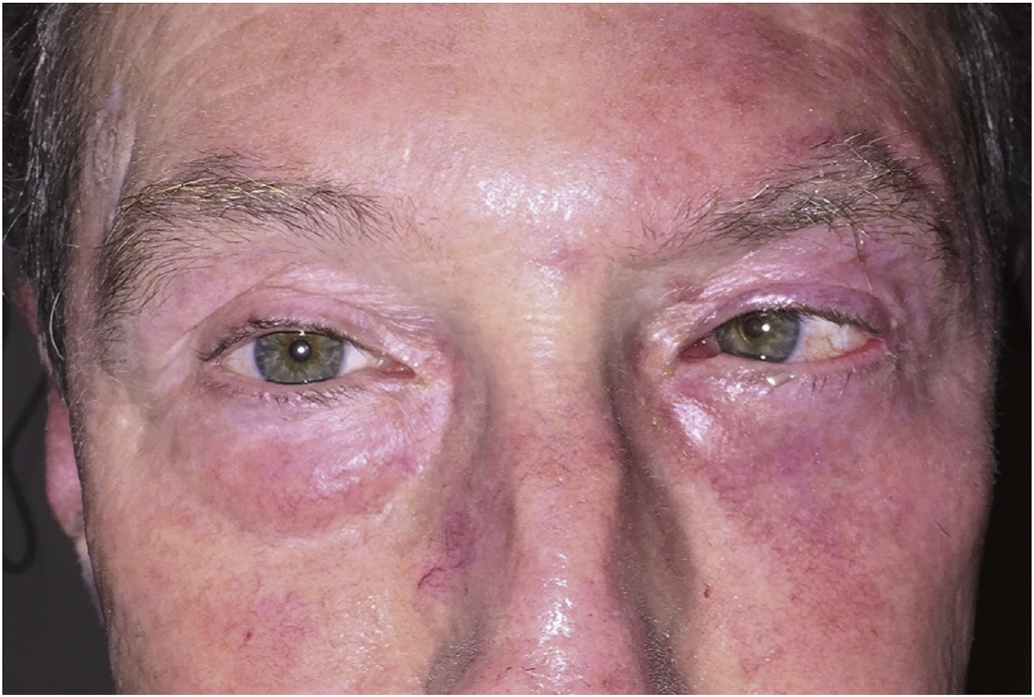
Clinical color photograph. A color photograph of the patient's face obtained after he had received radiation therapy for his diagnosis of recurrent basal cell carcinoma with perineural invasion. There is esotropia and no sign of a cutaneous malignancy, representative of his pre-treatment appearance. Erythema of the left eyelid and atrophy of the left inferior eyelid fat pad are related to radiation therapy. (For interpretation of the references to color in this figure legend, the reader is referred to the Web version of this article.)

A 62-year-old male was referred for 18 months of left forehead numbness, 9 months of horizontal binocular diplopia, and 3 months of left cheek numbness. He had a history of Mohs micrographic surgery (MMS) 9 years prior for a left eyebrow BCC. Though he lacked clinical neuropathies at that time, the BCC was infiltrative, ulcerated, and demonstrated histologic PNI. Therefore, Mohs excision extended into the frontalis muscle to obtain 3-mm tumor-free margins. On examination in our office 9 years later, the patient demonstrated a left cranial nerve VI palsy and hypoesthesia along the left V_1_ and V_2_ dermatomes. There were no suspicious skin lesions or lymphadenopathy (Fig. 1).

**Fig. 2:**
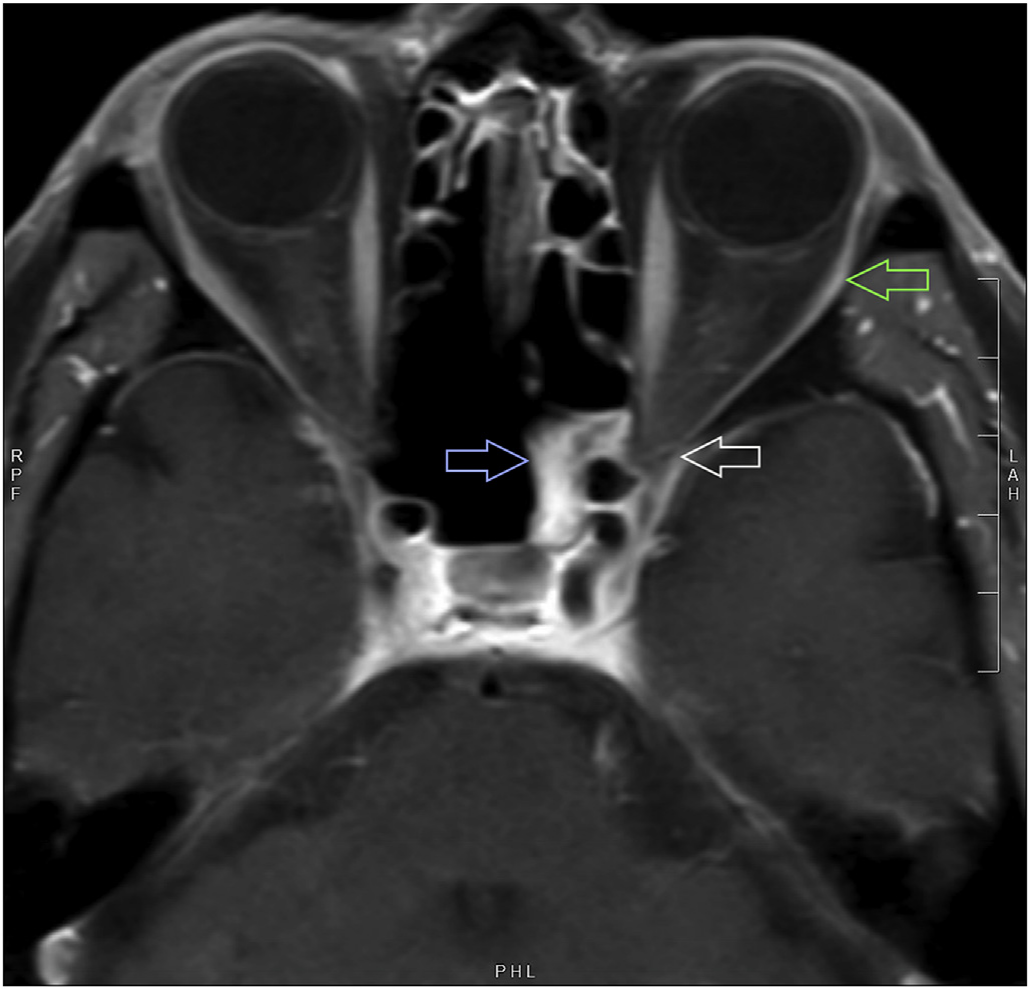
Magnetic resonance imaging. A T1-weighted, post-gadolinium, axial magnetic resonance imaging section of the brain and orbit showing left lateral rectus atrophy (green arrow), left sphenoid sinus opacification (blue arrow), and equivocal fullness of the superior orbital fissure (white arrow). (For interpretation of the references to color in this figure legend, the reader is referred to the Web version of this article.)

An extensive workup had been performed over the preceding 12 months prior to referral. Cholesterol, blood pressure, blood glucose, complete blood count, acetylcholine receptor binding and blocking antibodies, erythrocyte sedimentation rate, and C-reactive protein were unremarkable. Serial MRIs over the preceding seven months identified progressive atrophy of the left lateral rectus muscle without abnormality specific to the ophthalmic (V_1_) or maxillary (V_2_) branches of the left trigeminal nerve. Review by multiple neuro-radiologists and clinicians suggested that the left superior orbital fissure and left lateral cavernous sinus had either normal appearance or subtle fullness, lacking a clear consensus (Fig. 2). Imaging also revealed chronic opacification of the left sphenoid sinus. Endonasal biopsies of the sphenoid sinus showed chronic fungal sinusitis without invasive disease or necrosis. Cerebrospinal fluid cytology and whole-body PET/CT were negative for malignancy.

**Fig. 3:**
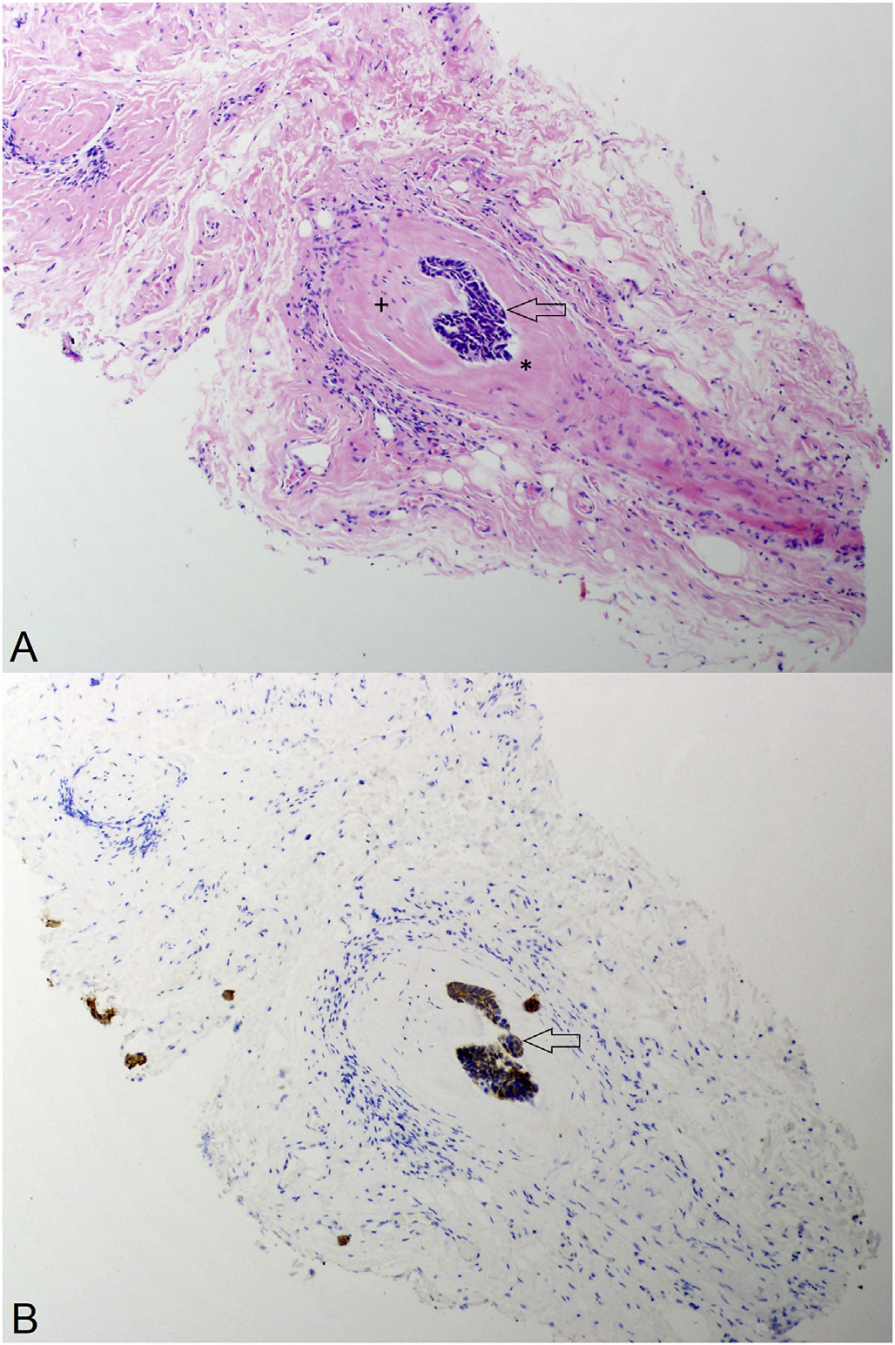
Histopathology. (A) A 10× magnification view of a pathologic tissue section from the supraorbital nerve biopsy, stained with hematoxylin and eosin. The section demonstrates a nest of invasive, epithelial cells with basaloid morphology including hyperchromatic nuclei without nucleoli and scant cytoplasm (arrow). There is adjacent S-100 positive neural tissue (+, stain not shown) residing within the perineurium (*), confirming tumor presence within the nerve. (B) A similar section stained with a cytokeratin cocktail to highlight the invasive cells. Further immunohistochemical testing (not shown) included p63 (positive), Ber-EP4 (positive), and EMA (negative), consistent with basal cell carcinoma.

PNI was suspected based on the patient's history of an ipsilateral BCC, and the patient underwent biopsy of the left supraorbital (V_1_) and infraorbital (V_2_) nerves via superior and inferior orbitotomies. Pathologic examination revealed normal infraorbital nerve tissue and PNI of the supraorbital nerve by an epithelial neoplasm with basaloid morphology (Fig. 3). Immunohistochemistry was consistent with BCC (pancytokeratin+, p63+, Ber-EP4+, EMA-). This pattern suggested retrograde spread of BCC along V_1_ into the cavernous sinus, followed by anterograde spread into cranial nerves V_2_ and VI, presumably with V_2_ involvement isolated to the region proximal of the negative biopsy site.

The patient was initiated on treatment for *American Joint Committee on Cancer* stage IV BCC, beginning with external beam radiation to the left orbit and cavernous sinus. He was placed on a continual course of vismodegib, a small molecule inhibitor of the hedgehog signaling pathway indicated for advanced basal cell carcinoma. After 6 months of treatment, he unfortunately required temporary cessation of his vismodegib due to adverse effects including weight loss, muscle spasm, loss of taste, and pruritic rash; however, at 10 months of follow-up, repeat magnetic resonance imaging showed no progressive disease. He recovered a small measure of abduction in the left eye, but ultimately required strabismus surgery to achieve orthotropia in primary gaze. His V_2_ paresthesia evolved into a persistent dysesthesia during treatment, while his V_1_ paresthesia remained static.

## 3 Discussion

This report describes a challenging case of symptomatic PNI caused by a recurrent BCC lacking specific radiographic or cutaneous abnormalities. PNI by BCC is a rare entity, with histologic evidence present in only 0.18–3.8% of BCCs,^5^, ^6^, ^7^ compared with 3–14% of squamous cell carcinomas.^2^ The prevalence of histologic PNI is greater for aggressive BCC subtypes, including the infiltrative subtype found in our case.^6^^,^^7^ A large series found that 7.2% of aggressive BCCs had histologic PNI, whereas less aggressive BCCs had PNI in 0.6% of cases.^7^ It is worth noting that only 30–40% of cases with histologically-confirmed PNI (by SCC or BCC) develop symptomatic cranial neuropathy.^1^

Our patient sequentially developed mixed cranial neuropathies, with V_1_, V_2_, and VI ultimately affected. Although the majority of patients with clinically-detectable PNI have only one cranial neuropathy, multiple nerves may become involved if the neoplasm spreads to a region of neural confluence,^3^^,^^4^ such as the cavernous sinus. Cranial nerve VII and V_2_ are most commonly affected due to their superficial branches in the mid-face, where most cutaneous malignancies occur, but involvement of every cranial nerve has been reported.^3^

This case demonstrates the high index of suspicion necessary to make a timely diagnosis of clinically significant PNI. Several factors can impede arrival at this diagnosis. First, symptoms insidiously develop over a median of six months prior to presentation.^4^ Second, while dermatologic examination is essential, a skin lesion is absent in nearly 25% of cases.^4^ Last, symptomatic PNI has been reported following seemingly clear-margin excision, fulguration or cryotherapy of presumed pre-malignant lesions, and uncommonly, in patients without a known history of a skin lesion.^3^^,^^4^ In cases of recurrent skin cancers, a median of 16 months (range 1–86) may separate primary carcinoma treatment and symptom onset.^4^ The latent period of 7.5 years (90 months) demonstrated in our case represents, to our knowledge, the longest reported interval between the diagnosis of primary BCC and biopsy-proven PNI by recurrent BCC, though for SCCs, the longest reported interval approaches two decades.^1^

Imaging studies are important for the diagnosis of clinically evident PNI, but must be ordered and interpreted with specific attention to the affected nerve(s). A propensity for subtle axial growth, rather than concentric growth, may cause PNI to be missed without careful review of imaging studies.^1^ Traditional, non-focused CT or MRI studies have sensitivity as low as 50% for symptomatic, histologically-proven PNI.^8^ Focused MRI should instead be pursued, involving thin slices along the course of the affected cranial nerve(s) with a sequence optimized for the suspected location of pathology. Neuro-radiologists possess several tools to enhance detection of PNI. MRI “neurography”, as originally described, is a protocol emphasizing ultra T2-weighted sequences with thin slices to offer excellent resolution of nerve tissue from cerebrospinal fluid. Notably, some authors and sites use the term more broadly to describe high resolution, focused cranial nerve MRI. Sommerville et al. described a sensitivity of 95–100% using this broader definition of neurography.^9^ Steady state precession sequences, more commonly known by trade names such as FIESTA (Fast Imaging Employing Steady-state Acquisition) or CISS (Constructive Interference Steady State), are another promising tool, though data is limited.^10^ These sequences derive signal intensity from the tissue ratio of T2 to T1 signal, again offering improved resolution between cerebrospinal fluid and nerve tissue.

In this case, neuro-radiology was most suspicious for pathology in the region of the superior orbital fissure, which involves discrimination of nerve from soft tissue. For this reason, they selected protocols emphasizing thinly-sliced T1-weighted sequences, rather than a steady state precession sequence or the ultra T2-weighted sequences of traditional neurography. Given the subtle radiologic signs of PNI, it is important for the clinician to communicate the expected etiology and locus of pathology to assist the neuro-radiologist in optimizing and focusing the protocol. The normal appearance of the histologically-involved V_1_ on high resolution, focused MRI in the present case is unusual. Subtle fullness of the superior orbital fissure and cavernous sinus was the only suggestive, albeit equivocal imaging finding. Biopsy of the clinically involved nerves should be pursued when PNI is suspected,^11^ even in the absence of a direct imaging correlate of the biopsy target.

The patient in this case had local histologic PNI associated with his primary BCC, and developed symptomatic PNI despite MMS with 3-mm tumor-free margins. There is no consensus on the appropriate management and surveillance of such high-risk patients with a primary cutaneous malignancy exhibiting histologic PNI. Measures including adjunctive radiotherapy, an additional stage of MMS, and surveillance imaging have been suggested, but data has been inconclusive.^1^^,^^5^^,^^12^ To reduce diagnostic delays, such patients should be counseled regarding symptoms of PNI, and practitioners should carry a high index of suspicion when encountering sequential development of cranial neuropathies or slowly progressive mononeuropathy during subsequent skin surveillance visits for years after treatment.

## 4 Conclusion

This report demonstrates the multitude of challenges faced in establishing a diagnosis of PNI by BCC. Not only is the disease process rare, but negative imaging studies and late presentation after uncomplicated treatment of the primary lesion can provide false reassurance.

## Patient consent

Written informed consent for publication of this report and all included images was obtained from the patient, and a copy is available for review by the journal editor.

## Funding

Open access article funding will be pursued from the UCSF Library Open Access Fund if the manuscript is accepted. The UCSF Library played no role in the writing of this manuscript.

## Conflicts of interest

The following authors have no financial disclosures: DCA, EKH, MHL, RCK.

## Authorship

All authors attest that they meet the current ICMJE criteria for Authorship.

## Disclosures

All procedures performed in this study involving a human participant adhered to the 1964 Declaration of Helsinki and its later amendments. Collection of information in this report complied with the Health Insurance Portability and Accountability Act of 1996.
