# Supplementary material for: Mixed cranial neuropathies due to occult perineural invasion of basal cell carcinoma
Source: Am J Ophthalmol Case Rep. 2018 Dec 19;13:136–9. doi: 10.1016/j.ajoc.2018.12.018 (PMC6348452; doi:10.1016/j.ajoc.2018.12.018)
Supplement: Multimedia component 1 [file mmc1.pdf]

Question 1:

What are the most common cranial nerves affected by perineural invasion of a maxillofacial cutaneous malignancy?

● VII and V2

Explanation:

Most cutaneous malignancies occur in the mid-face. Cranial nerves VII and V2 have superficial branches in this area, likely explaining their frequent involvement by PNI.

❍ V2 and III

❍ VI and V3

❍ VII and II

Question 2:

What portion of cases with perineural invasion will have a recurrent skin lesion on examination?

❍ 95%

❍ 10%

● 75%

Explanation:

A skin lesion was found to be absent in 25% of cases.

❍ 33%

Question 3:

What is the best imaging study to identify perineural invasion?

❍ MRI/MRA

❍ CT

❍ PET/CT

● MRI Neurography

Explanation:

High resolution MRI with thin cuts along the course of the clinically involved nerves provides a sensitivity of 95-100%.

Question 4:

What is the median interval between treatment of a cutaneous malignancy and development of cranial neuropathy due to recurrent disease with perineural invasion?

● 16 months

Explanation:

A large case series of patients with SCC found a median of 16 months had elapsed, but the range was 1 month to 86 months. A case report describes recurrence as late as two decades later for SCC, and the patient in this case report experienced symptoms 7.5 years after his BCC excision. Thus,the time elapsed since primary tumor treatment should not be thought to rule out perineural invasion as a cause of cranial neuropathy -- it must always be considered.

❍ 5 years

❍ 16 weeks

❍ 1 year
